# Validation of an albumin-indocyanine green-based China liver cancer staging system to evaluating resectable hepatocellular carcinoma patients and comparison with the Child-Pugh-based China liver cancer staging system

**DOI:** 10.3389/fonc.2025.1450333

**Published:** 2025-02-20

**Authors:** MinQiang Chen, Chao Ren, MengXia Wang, Min Yu, Bo Wu, Bo Zhuang, JianXiang Jin, YaoQi Zhang, ShiAn Yu

**Affiliations:** Department of Hepatobiliary and Pancreatic Surgery, Jinhua Municipal Central Hospital Medical Group, Jinhua, China

**Keywords:** albumin-indocyanine green, hepatocellular carcinoma, prognostic impact, china liver cancer staging, overall survival, recurrence-free survival

## Abstract

**Aim:**

Here, the utility of an albumin-indocyanine green-based China liver cancer (CNLC) staging system (ALICE-CNLC) as a tool for the prognostic assessment of hepatocellular carcinoma (HCC) patients was evaluated, comparing this system to the Child-Pugh score-based CNLC staging system.

**Methods:**

The cohort for this study included 331 patients with HCC who had undergone hepatectomy at Jinhua Municipal Central Hospital Medical Group in China from April 2012-June 2021 and had postoperative pathology-confirmed HCC. Kaplan-Meier survival curves were generated, with log-rank tests used to examine prognostic factors. Univariate and multivariate analyses were used for identification of outcome predictors using Cox proportional hazards regression.

**Results:**

The prediction of overall survival (OS) by the ALICE-CNLC system for patients with stage Ia disease was markedly better than that for patients with stage Ib and IIa disease (*P*=0.010, *P*=0.026), while the latter groups did not differ significantly (*P*=0.796). The ALICE-CNLC system predicted the 3-year recurrence-free survival (RFS) rates for patients with stage Ia, Ib, and IIa disease to be 50.4%, 47.7%, and 25%, respectively, with significant differences among the groups (*P=*0.033, *P*<0.001, and *P=*0.043). These results were similar to those of the CNLC staging system.The OS and RFS did not differ significantly between the same grades of patients evaluated with the ALICE-CNLC and CNLC staging systems.

**Conclusion:**

The ALICE-CNLC and CNLC staging systems did not show significant differences in predicting the prognosis of patients with HCC who have undergone hepatectomy.

## 1 Introduction

Liver cancer staging is immensely important to the appropriate selection of treatments for affected patients and the accurate assessment of their prognosis (1–4). Many reports have emphasized the value of utilizing liver cancer staging systems based on tumor-related symptoms, tumor burden, and hepatic function (5–9). The China liver cancer staging (CNLC) system, which is based on Child-Pugh scores, has provided value as a means of treatment planning and prognostic assessment, affording benefits to many patients (10, 11). Several non-invasive models for scoring liver function are currently used in clinical practice, including the Child-Pugh, albumin biliribin (ALBI) and albumin indocyanine green (ALICE) scores. While the Child-Pugh scoring system is widely used in clinical practice, it has well-known limitations (12–19), including the subjective assessment of the degree and scoring of ascites and hepatic encephalopathy, potentially leading to variation in the results in clinical practice. Secondly, there are correlations between parameters such as serum albumin and the severity of ascites. Thirdly, the fact that the five parameters have the same weight in the scoring reduces the overall objectivity of the scoring. To date, there have been no comparisons of the degree of reliance on each parameter in the scoring. The ALBI score is subdivided into three subgroups of 1/2/3, while the ALICE score is subdivided into four subgroups of 1/2a/2b/3. Numerous studies have shown that these subgroups are also associated with significant survival differences, indicating that ALICE is a superior measure for evaluating patient prognosis (20–23). Here, the performance of an ALICE score-based CNLC staging system (ALICE-CNLC) was evaluated in terms of predicting the prognosis of patients with hepatocellular carcinoma (HCC), comparing this system with the conventional Child-Pugh score-based CNLC system.

## 2 Materials and methods

The study cohort included patients with HCC who had undergone hepatectomy at Jinhua Municipal Central Hospital Medical Group in China from April 2012-June 2021 and had postoperative pathology-confirmed HCC. Patients were excluded if they exhibited severe concomitant portal hypertension (24–26), had undergone radiotherapy, intravenous chemotherapy, or molecular targeted therapy within 1 month prior to surgery (27–30), exhibited obstructive jaundice (22, 31), or had undergone intraoperative combined splenectomy. Patient data were retrospectively obtained from 331 HCC patients included in this study. Surgical indications for HCC patients in this study were defined based on the Chinese Diagnostic and Treatment Guidelines for Primary Liver Cancer (10). The definition of major hepatectomy was the resection of a minimum of three Couinaud segments of the liver (21, 22).

ALICE scores were determined as 0.663 × log_10_ICG R15 (%)-0.0718 × albumin (g/L) (20–23). These ALICE scores were stratified into grade 1 (linear predictor value ≤-2.20), grade 2a (>-2.20 to ≤-1.88), grade 2b (>-1.88 to ≤-1.39), and grade 3 (>-1.39). The definition of overall survival (OS) was the time between surgical resection and death or the most recent follow-up, while recurrence-free survival (RFS) represented the time between surgical resection and recurrence or the most recent follow-up. The completion of follow-up was December 2022. Postoperative recurrence was determined by clinical physicians based on CT/MRI and serum tumor markers during follow-up. In this study, we used two methods for the selection of cutoff values, namely, (1) the binary classification method, which selects the best cutoff value by calculation of the Jordan index, such as Operation time/Flood loss/Postal hospital time and (2) based on definitions in the literature, such as diameter.

Staging was performed as follows: (1) Stage Ia was defined by a solitary HCC tumor ≤ 5 cm in size with no invasion of the vasculature or extrahepatic expansion in patients with intact hepatic function (Child-Pugh A/B, ALICE 1/2a/2b) and a performance status (PS) of 0-2 points; (2) Stage Ib was defined by a solitary HCC tumor > 5 cm or multifocal HCC with 3 nodules or fewer, none larger than 3 cm in size, with no invasion of the vasculature or extrahepatic expansion in patients with intact hepatic function (Child-Pugh A/B, ALICE 1/2a/2b) and a PS of 0-2 points; (3) Stage IIa was defined by multifocal HCC with up to 3 nodules and a maximum diameter > 3 cm but with no evidence of vascular invasion or extrahepatic spread in patients with intact hepatic function (Child-Pugh A/B, ALICE 1/2a/2b) and a PS of 0-2 points. (4) Stage IIb was defined by multifocal HCC with at least 4 nodules with no evidence of vascular invasion or extrahepatic spread in patients with intact hepatic function (Child-Pugh A/B, ALICE 1/2a/2b) and a PS of 0-2 points. (5) Stage IIIa was defined by invasion of the vasculature with no extrahepatic expansion in patients with intact hepatic function (Child-Pugh A/B, ALICE 1/2a/2b) and a performance status (PS) score of 0-2 points. (6) Stage IIIb was defined as extrahepatic expansion and/or invasion of the vasculature in patients with intact hepatic function (Child-Pugh A/B, ALICE 1/2a/2b) and a PS score of 0-2 points. (7) Stage IV was defined as end stage liver function (Child-Pugh C, ALICE 3) and/or major cancer related symptoms (PS >2). The Ethics Committee of the institution approved this study, which was performed as per the Declaration of Helsinki.

SPSS 23.0 (IBM Corp., Armonk, NY, USA) was used for data analysis. Kaplan-Meier curves with log-rank tests were used for survival analysis. Cox proportional hazards regression was used for univariate and multivariate analyses aimed at identifying predictive factors. *P* < 0.05 was regarded as significant.

## 3 Results

### 3.1 Characteristics

The median age of the study participants was 59 years (range: 26-92), and 289 participants (87.3%) were male. Of these patients, 257 (77.6%) exhibited hepatitis B surface antigen positivity. These patients were classified using the CNLC staging system into those with stage Ia (n=237, 71.6%), Ib (n=65, 19.6%), IIa (n=28, 8.5%), and IV disease (n=1, 0.3%). Similarly, the ALICE-CNLC staging system was used to classify these patients into those with stage Ia (n=238, 71.9%), Ib (n=65, 19.6%), and IIa disease (n=28, 8.5%). Of these patients, 331 underwent follow-up for a median duration of 45 months (range: 1-129), with a loss-to-follow-up rate of 3.0% (10/331), and 53 deaths on completion of follow-up. In addition, there were 117 instances of recurrence (35.3%), 83 of intrahepatic recurrence, 7 of extrahepatic recurrence, and 27 of intrahepatic and extrahepatic recurrence.

### 3.2 Post-hepatectomy overall survival

**Figure 1:**
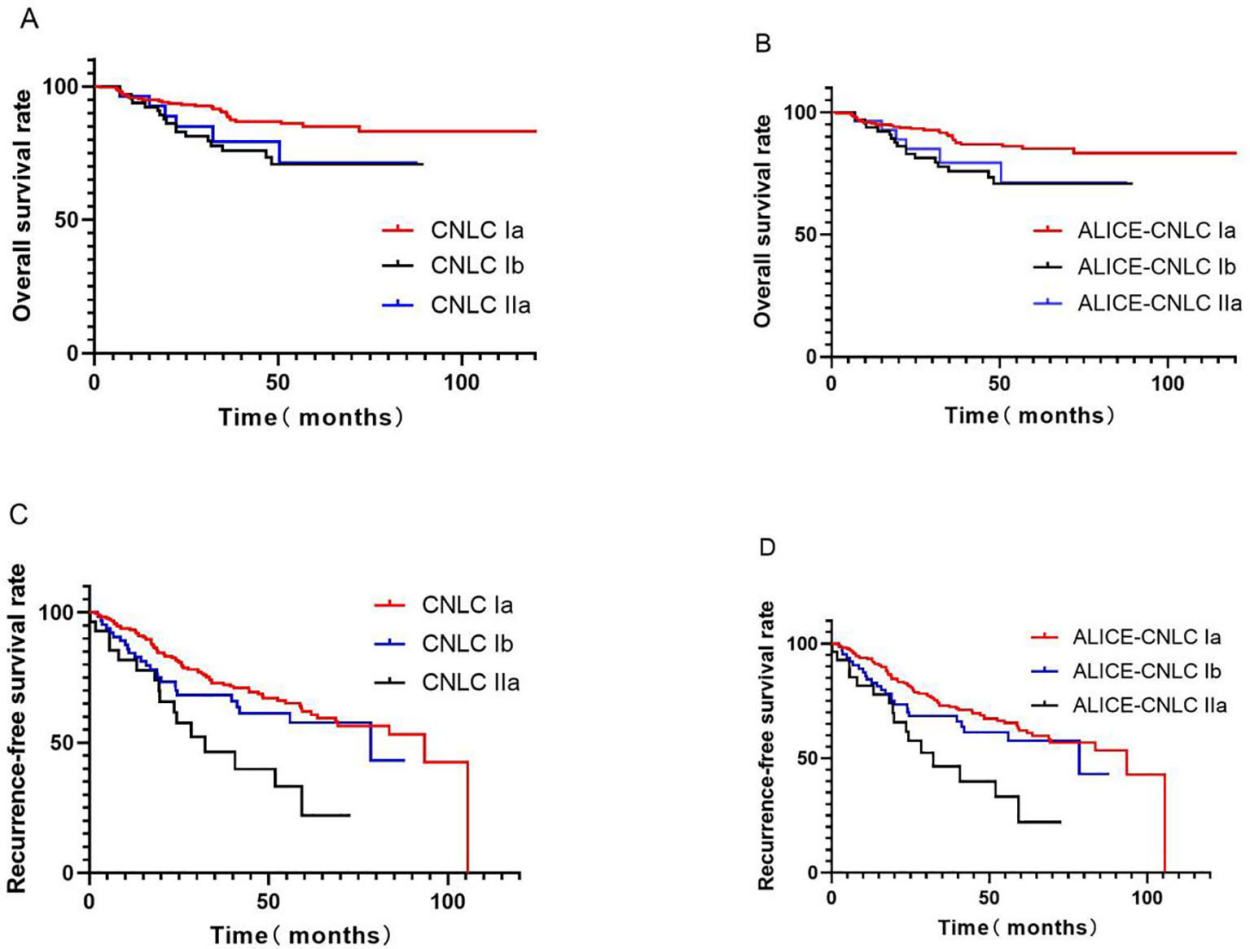
Survival curves for different staging systems. **(A)** OS using CNLC; **(B)** OS using ALICE-CNLC; **(C)** RFS using CNLC; **(D)** RFS using ALICE-CNLC.

Following hepatectomy, the median OS for the overall patient cohort was 45 months, with 1-, 3-, and 5-year OS rates of 94.9%, 61.9%, and 33.2%, respectively. With respect to the CNLC staging system, the corresponding 3-year OS values for those with stage Ia, Ib, and IIa disease were 64.3%, 60.0%, and 46.4%, respectively. Participants with stage Ia disease presented with significantly better prognostic outcomes than those with stage Ib and IIa (*P*=0.010, *P*=0.026), but the latter two groups did not differ significantly in terms of outcome (*P*=0.796). With respect to ALICE-CNLC staging system, the 3-year OS values for individuals with stage Ia, Ib, and IIa disease were 64.1%, 59.3%, and 44.4%, respectively, with the differences in prognosis among these stages being similar to those when using the CNLC staging system (*P=*0.009, *P=*0.023, and *P=*0.796), **Figure 1**.

### 3.3 Post-hepatectomy recurrence-free survival

Median RFS following hepatectomy in the overall patient cohort was 35 months, with respective RFS 1-, 3-, and 5-year values of 84.6%, 47.7%, and 22.4%. With respect to CNLC staging system, the recurrence-free 3-year survival for individuals with stage Ia, Ib, and IIa disease were 50.2%, 47.7%, and 25.0%, respectively. Prognostic outcomes for individuals with stage Ia disease were significantly better than those for stage Ib and IIa (*P*=0.046, *P*<0.001), with the same being true for stage Ib relative to stage IIa (*P*=0.043). With respect to the ALICE-CNLC staging system, the 3-year RFS values for stage Ia, Ib, and IIa patients were 50.4%, 47.7%, and 25%, respectively, with significant differences in prognosis across all grades (*P=*0.033, *P*<0.001 and *P=*0.043), **Figure 1**.

### 3.4 Independent predictors of overall survival

Next, factors associated with postoperative HCC patient OS were analyzed. In univariate analyses, the following were found to be predictors of OS: age > 65 years; AFP > 36.8 ng/mL; PT > 13 s; tumor diameter > 3 cm; blood loss > 275 mL; duration of postoperative hospitalization > 10 d; intraoperative ultrasound; major hepatectomy; microvascular invasion; perineural invasion; CNLC stage ≥ Ib; ALICE-CNLC stage ≥ Ib; BCLC stage ≥A; and ALICE-BCLC stage ≥ A(all *P*<0.05). Of these factors, high blood loss, perineural invasion, high CNLC stage, and high ALICE-CNLC stage all showed independent associations with patient OS (**Table 1**).

**Table 1:** Cox proportional hazards regression analyses of overall survival.

Risk factors	Univariate analysis		Multivariate analysis	
	HR (95% CI)	*P*	HR (95% CI)	*P*
Male sex	1.311 (0.640-2.688)	0.460		
Age >65 (year)	2.064 (1.007-4.228)	0.048	1.101 (0.635-1.987)	0.951
Viral hepatitis B	1.002 (0.464-1.753)	0.761		
AFP >36.8 (ng/mL)	2.243 (1.306-3.852)	0.003	1.832 (0.556-6.034)	0.319
Albumin ≤40 (g/L)	1.631 (0.940-2.830)	0.082		
T-bil >12 (μmol/L)	2.046 (0.924-4.533)	0.078		
PT >13 (s)	2.144 (1.249-3.682)	0.006	0.497 (0.098-2.511)	0.397
ICG R15 >6 (%)	1.036 (0.595-1.806)	0.900		
Ascites	1.095 (0.751-1.595)	0.637		
Tumor diameter >3 (cm)	2.370 (1.245-4.512)	0.009	7.379 (0.827-65.847)	0.073
Multiple	1.186 (0.507-2.755)	0.694		
Laparotomy	1.692 (0.979-2.926)	0.060		
Operation time >127 (min)	1.887 (0.971-3.665)	0.061		
Hepatic portal occlusion reperfusion	0.841 (0.489-1.449)	0.533		
Intraoperative ultrasound	2.730 (1.287-5.794)	0.009	6.093 (0.745-49.812)	0.092
Major hepatectomy	2.266 (1.229-4.176)	0.009	0.533 (0.125-2.272)	0.395
Blood loss >275 (ml)	3.181 (1.727-5.860)	<0.001	6.540 (1.444-29.616)	0.015
Duration of postoperative hospitalization > 10 d	1.811 (1.056-3.106)	0.031	1.250 (0.341-4.574)	0.736
Child-Pugh B grade	1.603 (0.390-6.589)	0.513		
ALICE 2b grade	0.891 (0.321-2.471)	0.825		
Cirrhosis	1.097 (0.627-1.917)	0.746		
Poorly differentiated	1.853 (0.753-4.559)	0.179		
Microvascular invasion	5.221 (1.216-22.419)	0.026	1.146 (0.124-10.605)	0.905
Perineural invasion	6.965 (2.331-20.816)	0.001	5.654 (1.832-17.444)	0.003
CNLC ≥ Ib stage	2.092 (1.215-3.602)	0.008	10.812 (1.010-115.790)	0.049
ALICE-CNLC ≥ Ib stage	2.104 (1.222-3.623)	0.007	10.245 (1.007-114.765)	0.048
BCLC ≥ A stage	2.553 (1.016-6.416)	0.046	1.372 (0.545-2.679)	0.931
ALICE-BCLC ≥ A stage	2.538 (1.010-6.379)	0.048	1.372 (0.545-2.679)	0.931

HR, hazard ratio; CI, confidence interval; AFP, alpha-fetoprotein; T-bil, total bilirubin; PT, prothrombin time activation rate; ICG R15, indocyanine green retention rate after 15 min; ALICE, Albumin-Indocyanine Green Evaluation; CNLC, China Liver Cancer Staging; BCLC, Barcelona Clinic Liver Cancer Staging; ALICE-CNLC, the China liver cancer staging based on the ALICE score; ALICE-BCLC, Barcelona Clinic Liver Cancer Staging based on the ALICE score.

### 3.5 Independent predictors of recurrence-free survival

Factors associated with RFS in postoperative HCC patients were also analyzed. In univariate analyses, the following were found to predict RFS: AFP > 36.8 ng/mL; albumin ≤40 g/L; tumor diameter > 3 cm; the presence of multiple tumors; operation time > 127 min; major hepatectomy; blood loss > 275 mL; duration of postoperative hospitalization > 6.5 d; CNLC stage ≥ IIa; ALICE-CNLC stage ≥ IIa; BCLC stage ≥ A; and ALICE-BCLC stage ≥ A (all *P*<0.05). OF these factors, high AFP, albumin levels, tumor diameter, high blood loss, high CNLC stage, high ALICE-CNLC stage, high BCLC stage, and high ALICE-BCLC stage were all found to be independently linked with RFS (**Table 2**).

**Table 2:** Cox proportional hazards regression analyses of recurrence free survival.

Risk factors	Univariate analysis		Multivariate analysis	
	HR (95% CI)	*P*	HR (95% CI)	*P*
Male sex	1.112 (0.645-1.916)	0.703		
Age >65 (year)	1.273 (0.826-1.962)	0.274		
Viral hepatitis B	1.012 (0.510-1.593)	0.380		
AFP >36.8 (ng/mL)	1.474 (1.022-2.125)	0.038	1.578 (1.094-2.276)	0.015
Albumin ≤40 (g/L)	1.499 (1.035-2.173)	0.032	1.456 (1.004-2.111)	0.048
T-bil >12 (μmol/L)	1.028 (0.667-1.583)	0.550		
PT >13 (s)	1.235 (0.827-1.845)	0.303		
ICG R15 >6 (%)	1.410 (0.974-2.041)	0.069		
Ascites	1.097 (0.842-1.430)	0.493		
Tumor diameter >3 (cm)	2.343 (1.535-3.578)	<0.001	2.152 (1.401-3.303)	<0.001
Multiple	1.860 (1.109-3.121)	0.019	0.508 (0.069-3.772)	0.508
Laparotomy	0.915 (0.634-1.320)	0.635		
Operation time >127 (min)	1.653 (1.079-2.532)	0.021	1.137 (0.703-1.838)	0.601
Hepatic portal occlusion reperfusion	0.979 (0.812-1.181)	0.827		
Intraoperative ultrasound	0.943 (0.779-1.142)	0.550		
Major hepatectomy	1.654 (1.046-2.613)	0.031		
Blood loss >275 (ml)	1.767 (1.215-2.571)	0.003	1.578 (1.079-2.307)	0.019
Duration of postoperative hospitalization > 6.5 d	1.893 (1.129-3.175)	0.016	1.181 (0.675-2.065)	0.560
Child-Pugh grade	2.398 (0.761-7.552)	0.135		
ALICE 2b grade	1.430 (0.784-2.607)	0.243		
Cirrhosis	1.268 (0.867-1.856)	0.221		
Poorly differentiated	1.214 (0.658-2.239)	0.534		
Microvascular invasion	1.760 (0.970-3.195)	0.063		
Perineural invasion	2.204 (0.992-4.895)	0.052		
CNLC ≥ IIa stage	2.405 (1.414-4.092)	0.001	1.698 (0.973-2.963)	0.049
ALICE-CNLC ≥ IIa stage	2.418 (1.421-4.114)	0.001	1.678 (0.969-2.873)	0.049
BCLC ≥ A stage	2.492 (1.389-4.472)	0.002	2.033 (1.118-3.697)	0.020
ALICE-BCLC ≥ A stage	2.474 (1.379-4.440)	0.002	2.033 (1.118-3.697)	0.020

HR, hazard ratio; CI, confidence interval; AFP, alpha-fetoprotein; T-bil, total bilirubin; PT, prothrombin time activation rate; ICG R15, indocyanine green retention rate after 15 min; ALICE, Albumin-Indocyanine Green Evaluation; CNLC, China Liver Cancer Staging; BCLC, Barcelona Clinic Liver Cancer Staging; ALICE-CNLC, the China liver cancer staging based on the ALICE score; ALICE-BCLC, Barcelona Clinic Liver Cancer Staging based on the ALICE score.

### 3.6 Differences in matching grades from different staging systems

Comparison of the OS of patients with the same grade of HCC across these staging systems showed that OS did not differ significantly between ALICE-CNLC and CNLC stage Ia participants (*P=*0.981), between ALICE-CNLC and CNLC stage Ib disease (*P=*0.960), or between ALICE-CNLC and CNLC stage IIa disease (*P=*0.978). Similarly, the RFS did not differ significantly when comparing the same grades of the ALICE-CNLC and CNLC staging systems (*P=*0.963, *P=*0.989, and *P=*0.997).

## 4 Discussion

Indocyanine Green (ICG) has become one of the most widely used fluorophores in clinical surgery due to its well-established applications and low rates of toxicity and allergic reactions (32–34). ICG fluorescence-guided surgery (FGS), an intraoperative imaging system, enhances intraoperative navigation and decision-making during the surgical procedure, benefiting a significant number of patients with HCC (35–37). ICG also plays a key role in liver function assessment. In 2016, Kokudo et al. (23) used data from a multi-institutional international database on patients (n=1868) who underwent liver resection to identify independent factors affecting the survival and postoperative outcomes of patients with resectable HCC. Considering the influence of the ALBI score, they limited the influencing factors to albumin and ICG R15, and developed the ALICE score via a randomly assigned training cohort, to make this model more concise and practical. ALICE scores have provided an objective, simple, and sensitive means of assessing hepatic function (20–22). The CNLC staging system is among the most common systems used to stage liver cancer. A CNLC staging system that incorporates this new analytical approach (ALICE) is thus a logical step forward.

The present analyses revealed that the ALICE-CNLC system exhibited good discriminative performance as a predictor of HCC patient OS and RFS. With increasing disease staging, patient survival tends to decrease, offering discriminative performance similar to that of the Child-Pugh score-based CNLC system. When the same stages were compared across scoring systems, no significant differences were detected. There may be several reasons for this: (1) Both the ALICE and Child-Pugh scores are sensitive indicators for assessing liver function, and in this study, the proportion of individuals with good liver function was relatively high, thus obscuring the ability of the ALICE score to distinguish liver function. We are considering expanding our target population for evaluating ALICE-CNLC to include individuals with reduced liver function or those receiving immunotherapy or molecular-targeted therapy. We also intend to evaluate the difference between the ALICE and Child-Pugh scores in individuals with more severe tumor burdens, such as the presence of tumors larger than 5 cm or more than 3 tumors, which may be better able to distinguish the differences. (2) The tumor related symptoms and tumor burst of CNLC staging and ALICE-CNLC staging are the same, which greatly affects the difference between them.

No significant differences Between CNLC and ALICE-CNLC were observed in this study, which at least indicates that the use of the ALICE score instead of the Child-Pugh score is feasible in HCC patients with good liver function who are undergoing liver resection. The Child-Pugh score evaluates liver function by assigning scores to five parameters. For patients with good liver function (5-6 points), further subdivision is impossible, especially in those with good liver function or liver cirrhosis with compensatory liver function (38–40). If patients undergo different treatments, such as local resection or extensive liver resection, this may have a serious impact on postoperative outcomes. Meanwhile, the Child-Pugh score cannot monitor changes in liver function during treatment, while ALICE is more sensitive, and fluctuations in the score can fully capture changes in liver function, thereby guiding timely changes in treatment to prevent serious adverse outcomes. The ALICE score involves only two parameters, which is convenient in practical applications. This differs from the five parameters used for the determination of the Child-Pugh score, and the use of the ALICE score thus reduces the economic and medical burden incurred by the extra evaluations, which is more important in resource-limited environments. In addition, compared to the subjectivity of the Child-Pugh scoring and the correlation between indicators, the ALICE scoring is more objective (20–23).

This study is subject to certain limitations. For one, this study included one individual with CNLC stage IV disease, which was categorized as stage Ia disease with the new ALICE-CNLC scoring system. This patient, who had significant loss of liver function, low albumin, and a small amount of ascites, was carefully observed. The ALICE score was 2b, and good recovery was achieved through local liver resection. This demonstrates that the ALICE score was better able to distinguish individuals with liver dysfunction, thanks to the accurate calculation resulting from statistical models, which compensates for the loss of evaluation capability caused to a certain extent by the scoring system. Unfortunately, we did not analyze any more samples and thus cannot yet rule out bias. The patient underwent local resection of the liver tumor, which had minimal impact on liver function. This may also be the main reason for the unchanged postoperative prognosis.

Secondly, this study only assessed the prognostic outcomes of those HCC patients who underwent surgery, focusing these analyses on subjects with stage Ia, Ib, and IIa disease. While ALICE-CNLC performs well in HCC patients undergoing liver resection, the clinical characteristics of patients with advanced HCC are more complex, and often involve significantly reduced liver function, increased tumor burden due to vascular invasion or a greater number of tumors, and a diversity of treatment options. The accurate staging of tumors and accurate assessment of liver function will benefit patients. We hope to fully evaluate the applicability of ALICE-CNLC in the late-stage HCC population receiving immune/molecular-targeted therapy in the future, and assess their ALICE scores or value of the ALICE-CNLC score during comprehensive treatment, especially in relation to changes in treatment.

We have previously evaluated the specific value of using BCLC staging based on the ALICE score. Firstly, we must acknowledge that there are certain similarities between CNLC and BCLC staging. There are some differences between them: (1) Compared to BCLC staging, CNLC staging is more detailed and has more relaxed requirements in terms of tumor size and PS score. BCLC staging is divided into 0/A/B/C/D, while CNLC is divided into Ia/Ib/IIa/IIb/IIIa/IIIb/IV. In practical applications, we have found that the more detailed staging is helpful in distinguishing some specific patients. For example, while some single giant tumors are classified as BCLC A in the BCLC staging system, they can be more finely distinguished in CNLC staging, providing greater guidance for subsequent treatment strategies (6, 10); (2) The recommended treatment methods vary depending on the stage. For example, surgery is not the first choice for BCLC B-grade patients. In Chinese staging, if the tumor is confined to the same segment or ipsilateral liver in CNLC IIb staging, R0 resection can be performed, or if R0 resection is performed in combination with portal vein thrombectomy in stage IIIa, surgical resection is also recommended. This method of differentiation is completely different from that used with BCLC, and practice has proven that a large number of Chinese patients benefit from it. However, we have also observed that the recommended treatment methods in BCLC staging are more detailed and the screening criteria are more comprehensive. Thus, while the two staging systems are similar, there are many differences, and whether ALICE-CNLC can be used as a supplement to BCLC in the future requires further research, especially in Western populations.

In summary, the ALICE-CNLC and CNLC staging systems did not show significant differences in predicting the prognosis of patients with HCC who have undergone hepatectomy.

## Data Availability

The original contributions presented in the study are included in the article/supplementary material. Further inquiries can be directed to the corresponding author.
